# Histamine: a potential cytoprotective agent to improve cancer therapy?

**DOI:** 10.1038/cddis.2015.378

**Published:** 2015-12-31

**Authors:** D J Martinel Lamas, M B Nicoud, H A Sterle, G A Cremaschi, V A Medina

**Affiliations:** 1 Laboratory of Radioisotopes, School of Pharmacy and Biochemistry, University of Buenos Aires, Buenos Aires, Argentina; 2 Laboratory of Cellular and Molecular Biology, Institute for Biomedical Research (BIOMED), School of Medical Sciences, Pontifical Catholic University of Argentina (UCA), and the National Scientific and Technical Research Council (CONICET), Buenos Aires, Argentina; 3 Neuroimmunomodulation and Molecular Oncology Division, Institute for Biomedical Research (BIOMED), School of Medical Sciences, Pontifical Catholic University of Argentina (UCA), and the National Scientific and Technical Research Council (CONICET), Buenos Aires, Argentina

Chemotherapy along with radiotherapy is a major treatment of medical oncology. Despite their therapeutic effects achieving local tumor control and in many cases controlling metastasis, both approaches can produce serious adverse effects to normal tissues either immediately or during the long term after treatment. Thus, their related toxicity frequently outweigh clinical benefits and worsen patient's quality of life.^1, 2^

Regardless of the high incidence of cancer therapy-associated adverse reactions (e.g., hepato, hemato and cardio toxicities) and their significant impact on morbidity, mortality and health economics; as far as we know, there are no standard, clinically approved, effective agents that could improve the therapeutic index of chemo and radiotherapy, controlling these devastating side effects in patients undergoing anti-tumoral treatments.^1, 2^ Therefore, the development of pharmacological approaches to effectively prevent conventional cancer therapy-induced toxicity is of utmost importance.

In this line, there are several reports demonstrating that histamine could be a promising selective pharmacological agent for the protection of radio-sensitive healthy tissues against ionizing radiation-induced adverse effects. Histamine administration was safely used in different radiobiological experimental models and produced a marked preservation of gamma radiation impaired morphological and functional characteristics of small intestine, salivary glands and bone marrow.^3, 4, 5^ Histamine has also demonstrated efficacy in ameliorating boron neutron capture therapy-induced mucositis in an oral pre-cancer model.^6^

It is important to highlight that histamine exerts anti-proliferative effects in different experimental models of human breast cancer, melanoma and cholangiocarcinoma.^7, 8^ Even more, histamine potentiates radiation-induced anti-neoplastic effects in human breast cancer cells. Recent results demonstrated that the combined treatment of histamine and gamma radiation leads to enhanced apoptosis, senescence and DNA damage, modulating oxidative stress. In addition, the *in vivo* treatment with histamine significantly reduced the size and the increased exponential doubling time of irradiated triple-negative breast tumors (TNBT) induced in nude mice with MDA-MB-231 cells.^9^

Breast cancer is the most common cause of cancer death in women worldwide. The anthracycline doxorubicin is one of the standards of care in TNBT, which accounts for 15–20% of all breast cancers, and is characterized by poor prognosis.^10^ In addition, doxorubicin is a highly effective anti-neoplastic agent, being one of the most commonly systemic treatments to improve other several adult and pediatric cancers, including both hematological and solid tumors.^2^ Unfortunately, the clinical efficacy of doxorubicin is hindered by dose-related adverse effects that could be acute or become evident years after finalizing chemotherapy.^2^ Doxorubicin-induced toxicities include hematopoietic suppression, hepatotoxicity and life-threatening cardiotoxicity, being the latter the most serious doxorubicin-related side effect.^2, 11^

Numerous doxorubicin-induced cytotoxic mechanisms have been identified. Like gamma radiation, doxorubicin induces in part its anti-proliferative effects by augmenting reactive oxygen species and inducing DNA damage.^2^ Hence, oxidative stress has a key role in both doxorubicin-induced cardiac and hepatic toxicities.^2, 11^ Unfortunately, there are no clinically effective therapeutics for doxorubicin-associated cardio or hepatotoxicity.

Recently, Martinel Lamas *et al.*^12^ demonstrated the cytoprotective capacity of histamine against doxorubicin-induced injury of normal tissues in different pre-clinical experimental models. The authors evaluated the effect of histamine administration on doxorubicin-induced hepatic and cardiac toxicity in rat and mouse species and in a TNBT-bearing mouse model, exploring histopathological, oxidative stress and biochemical parameters.^12^

This study,^12^ shows that pre-treatment with histamine prevented doxorubicin-induced cardiac toxicity, producing a significant preservation of the myocardium integrity of both Sprague–Dawley rats and Balb/c mice, more likely through a reduction of doxorubicin-induced oxidative stress and DNA damage. Histamine significantly alleviated histological damage, reducing lipid peroxidation, serum creatine kinase-myocardial band activity and expression of DNA double-strand breaks marker *γ*H2AX. Histamine also blocked the decrease in heart weight, whereas it enhanced thiol levels in doxorubicin-treated animals (Figure 1). These findings are in agreement with previous works, which demonstrated the protective effect of histamine on radiation-induced damage through the modulation of antioxidant enzymes and the reduction of genotoxic damage.^3, 4^

To further support the protective role of histamine, it was recently reported that histamine deficiency aggravated myocardial damage in acute myocardial infarction through impaired macrophage infiltration and increased apoptosis of cardiomyocytes.^13^

In their study,^12^ the authors also showed that histamine administration prevented doxorubicin-induced hepatotoxicity, which is another common complication of doxorubicin chemotherapy.^11^ Pre-treatment with histamine mitigated hepatocyte apoptosis and liver atrophy in an oxidative stress setting (Figure 1). In addition, histamine blocked doxorubicin-induced reduction of Kupffer cells, long-lived liver macrophages, in accordance with the study by Deng *et al.*^13^ that indicated the importance of histamine in modulating the infiltration and differentiation of macrophages in damaged areas, which contribute to tissue healing. Also, other authors reported that histamine effectively protects liver against I/R-induced histologic, functional and oxidative damage.^8^

The combination therapy with doxorubicin and histamine was further evaluated in TNBT to verify whether it could compromise chemotherapeutic efficacy. Histamine revealed synergistic anti-tumor activity when administered with doxorubicin both *in vitro* and *in vivo* models of TNBT, enhancing doxorubicin-induced apoptosis and DNA damage (Figure 1). Remarkably, the mice bearing TNBT further verified the selective cardio and hepatoprotective action of histamine.

On the basis of these findings (Figure 1), Martinel Lamas *et al.*^12^ postulate that histamine exhibits chemoprotective effects against doxorubicin-induced cytotoxic and oxidative damage in heart and liver, without compromising the anti-tumor activity of doxorubicin. Thereby, the combined use of histamine with doxorubicin could be an attractive strategy to improve the therapeutic ratio of doxorubicin chemotherapy.

In the last decade, numerous clinical trials evaluated the effects of the combination of immunotherapy with interleukin (IL)-2 and histamine dihydrochloride (subcutaneous), for the potential treatment of metastatic melanoma, acute myelogenous leukemia and renal cell carcinoma.^14^ In agreement with Martinel Lamas *et al.*^12^ histamine dihydrochloride displayed enhanced anti-tumor activity when administered with IL-2 in the treatment of diverse types of tumors and has the potential to prevent oxidative stress-induced damage, preserving immune cells such as natural killer and T-cells.^14^

The presented evidences suggest that the promising combination therapeutic strategy with doxorubicin and histamine could be easily translated into clinical practice. Furthermore, a multifunctional targeting delivery system for doxorubicin that includes histamine in the micelle has been recently reported. This system facilitated the anti-tumor efficacy of doxorubicin in multidrug resistant breast cancer cells, through the enhancement of doxorubicin release in the tumor site and reducing its heart's uptake.^15^ Thus, the combination therapy based on doxorubicin-loading systems that include histamine could further improve therapeutic outcomes of TNBT.

In conclusion, the study by Martinel Lamas *et al.*^12^ suggests that cancer combination therapy with histamine could enhance clinical efficacy by reducing toxicity and potentiating anti-tumor activity and thereby improving patients' health and quality of life.

## Figures and Tables

**Figure 1:**
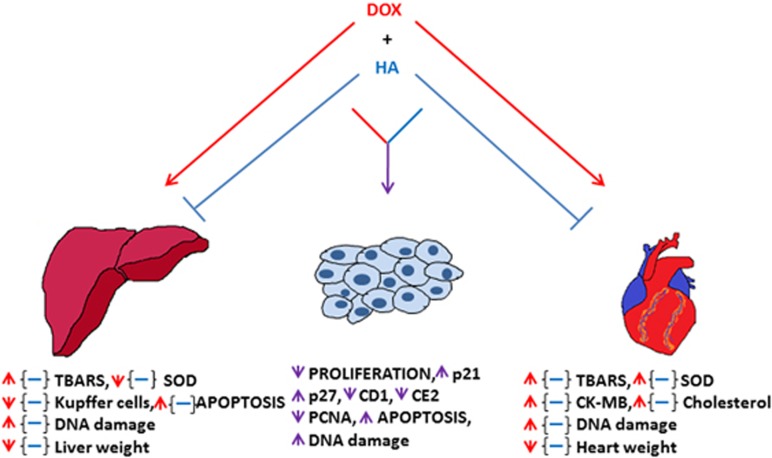
Histamine (HA) selective cytoprotective effect against doxorubicin (DOX) toxicity. HA blocked DOX-induced cytotoxicity in liver and heart mainly through the modulation of ROS and reducing DNA damage. In tumor cells, HA synergized DOX effects, inhibiting proliferation, augmenting DNA damage and apoptosis. Red arrows indicate DOX effects; blue dashes indicate HA blockade of DOX effects, and violet arrows indicate HA potentiation of DOX effects
